# Epidemiology of Road Traffic Mortalities among bus / minibus users in East Azerbaijan, Iran 

**Published:** 2019-08

**Authors:** Homayoun Sadeghi-Bazargani, Bahram Samadirad, Mina Golestani, Nasrin Shahedifar, Milad Jamali

**Affiliations:** ^*a*^Road Traffic Injury Research Center, Statistics and Epidemiology Department, Tabriz University of Medical Sciences, Tabriz, Iran.; ^*b*^Legal Medicine Research Center, Legal Medicine Organization, Tehran, Iran.; ^*c*^Tabriz International Safe Community Support Center, Tabriz, Iran.; ^*d*^Statistics and Epidemiology Department, Tabriz University of Medical Sciences, Tabriz, Iran.

## Abstract

**Background:**

The fatalities risk for passengers of cars, buses and minibuses are at least twice as large as ‎truckers. In Iran, according to the Forensic Medicine Database, over a 5-year period, 13 bus-‎related collisions lead to 919 deaths. Objective: The purpose of this study was to evaluate the ‎epidemiological characteristics of mortality due to road deaths in buses and minibuses during ‎‎2006 -2017 in East Azerbaijan Province of Iran. ‎

**Methods:**

The mortality rate of users of 245 buses / minibuses, recorded at the forensic medicine ‎databases were analyzed using STATA 13 statistical software. ‎

**Results:**

Men (70%) were assigned the majority of victims (mean age of 18.6 ± 41.5 years), after which ‎adults (79.18%), illiterate (22.4%) and self-employed (25.3%) were victims. Passersby and ‎police have played roughly zero roles in the transportation of victims since 2014. The process of ‎reducing of trending for bus and minibus users was observed over the study period. Head-neck-‎face trauma was more prevalent among those who died before admission. Rollover and falling ‎were significantly common among bus users and users of the minibus respectively. Trucks, vans ‎and trailers as crash counterpart vehicles caused 59% of deaths, with the exception of cases ‎where no other vehicle was involved. Victims were more at risk of death in the hospital, when ‎accidents occurred on the city’s inner roads (OR: 4.17; 95% CI: 1.7-9.9). The elderly have died ‎‎2.78 times more than patients in the hospital compared with other age groups (95% CI: 1.23-‎‎6.26). ‎

**Conclusions:**

To intervene on traffic-related knowledge need to be prioritized of attitudes and behaviors, ‎male adults, illiterate, and self-employed bus / minibuses users. The type of vehicle involved in ‎an accident should be considered as an important factor in the deaths of accidents. Further ‎research is needed in the future in this regard.‎

**Keywords:**

Epidemiology, Injuries, Mortality, Bus, Minibus, Road traffic accident

